# Electronic Metamaterials with Tunable Second-order Optical Nonlinearities

**DOI:** 10.1038/s41598-017-10304-2

**Published:** 2017-08-30

**Authors:** Hung-Hsi Lin, Felipe Vallini, Mu-Han Yang, Rajat Sharma, Matthew W. Puckett, Sergio Montoya, Christian D. Wurm, Eric E. Fullerton, Yeshaiahu Fainman

**Affiliations:** 1 Materials Science and Engineering, University of California, San Diego, 9500 Gilman Drive, La Jolla, California, 92093 USA; 2 Department of Electrical & Computer Engineering, University of California, San Diego, 9500 Gilman Drive, La Jolla, CA 92093 USA; 3 Center for Memory and Recording Research, University of California, San Diego, 9500 Gilman Drive, La Jolla, CA 92093-0401 USA

## Abstract

The ability to engineer metamaterials with tunable nonlinear optical properties is crucial for nonlinear optics. Traditionally, metals have been employed to enhance nonlinear optical interactions through field localization. Here, inspired by the electronic properties of materials, we introduce and demonstrate experimentally an asymmetric metal-semiconductor-metal (MSM) metamaterial that exhibits a large and electronically tunable effective second-order optical susceptibility (χ^(2)^). The induced χ^(2)^ originates from the interaction between the third-order optical susceptibility of the semiconductor (χ^(3)^) with the engineered internal electric field resulting from the two metals possessing dissimilar work function at its interfaces. We demonstrate a five times larger second-harmonic intensity from the MSM metamaterial, compared to contributions from its constituents with electrically tunable nonlinear coefficient ranging from 2.8 to 15.6 pm/V. Spatial patterning of one of the metals on the semiconductor demonstrates tunable nonlinear diffraction, paving the way for all-optical spatial signal processing with space-invariant and -variant nonlinear impulse response.

## Introduction

Materials with large second-order nonlinear optical susceptibility (χ^(2)^) (e.g., crystals of lithium niobate (LiNbO_3_) and potassium dihydrogen phosphate (KDP)) have been widely used for the realization of nonlinear optical functionalities including light modulation, switching and wave mixing^1–4^. However, integration of these materials with CMOS compatible photonic integrated circuits (PIC) remains challenging^5, 6^. CMOS compatible materials are either amorphous (e.g., SiO_2_, HfO_2_ and ZrO_2_, etc.)^7, 8^ or crystalline with a centrosymmetric diamond lattice structure (e.g., Si) and, consequently, do not possess second-order nonlinear optical susceptibility, χ^(2)^ ^9^. Strain engineering of crystalline silicon waveguides has been proposed and demonstrated as a promising mean to create a non-zero effective χ^(2)^, however, the magnitude of the strain induced χ^(2)^, has recently been shown to be much lower than that reported in the past (i.e., 8 ± 3 pm/V)^9–13^. The electric field induced second-harmonic (EFISH) generation introduces an effective χ^(2)^ by the interaction of an externally applied electric field with the material’s bulk third-order nonlinear susceptibility, χ^(3)^ ^14^. Nevertheless due to shielding of the external field by a semiconductor, a sufficiently high internal electric field exists only within the first tens of nanometers from the semiconductor interfaces for both metal-oxide-semiconductor (MOS) and metal-semiconductor (MS) structures^15–17^. Current research in creating CMOS compatible materials exhibiting a high χ^(2)^ coefficient mostly focuses on back-end processing^18^, composite metamaterials that utilize alternative physical mechanisms to synthesize non-zero χ^(2)^ ^19, 20^, metallo-dielectric multilayers^21^, dielectric-semiconductor multilayers^22^, all-dielectric composite metamaterials^8, 23^, and nonlinear processes enhanced by plasmonic metasurfaces and nanoatennas^24, 25^. However these approaches do not allow for a good control over the exhibited nonlinearities.

In this manuscript, we propose, systematically analyze and demonstrate experimentally an asymmetrical metal-semiconductor-metal (MSM) electronic metamaterial that exhibits a prominent and tunable effective χ^(2)^. The large induced χ^(2)^ originates from the interaction between the bulk third-order nonlinear optical susceptibility of the semiconductor, χ^(3)^ with the engineered non-zero net internal electric field created by the electronic transport close to the semiconductor interfaces clad with metals possessing dissimilar work functions. The MSM structure, consisting of CMOS-compatible amorphous silicon (a-Si) layer clad with aluminum (Al) and nickel (Ni), is shown experimentally to generate a second-harmonic field intensity five times larger than that from its constituents alone. Additionally, the nonlinear response of MSM is shown to be both proportional to the difference in the work functions of the cladding metals and actively controllable by an external electric field. The resulting effective *χ* ^*(2)*^_*zzz*_ tensor component ranges in value from 2.8 to 15.6 pm/V, making these structures suitable for on-chip optical switching and modulation. Moreover, by structuring one of the metals on the interface with a-Si and varying the externally applied electric field, we demonstrate tunable nonlinear diffraction effect, making it suitable for all-optical spatial signal processing with tailored space invariant as well as space variant nonlinear spatial impulse response.

## Results

### Analysis of the electronic nonlinear metamaterial

**Figure 1:**
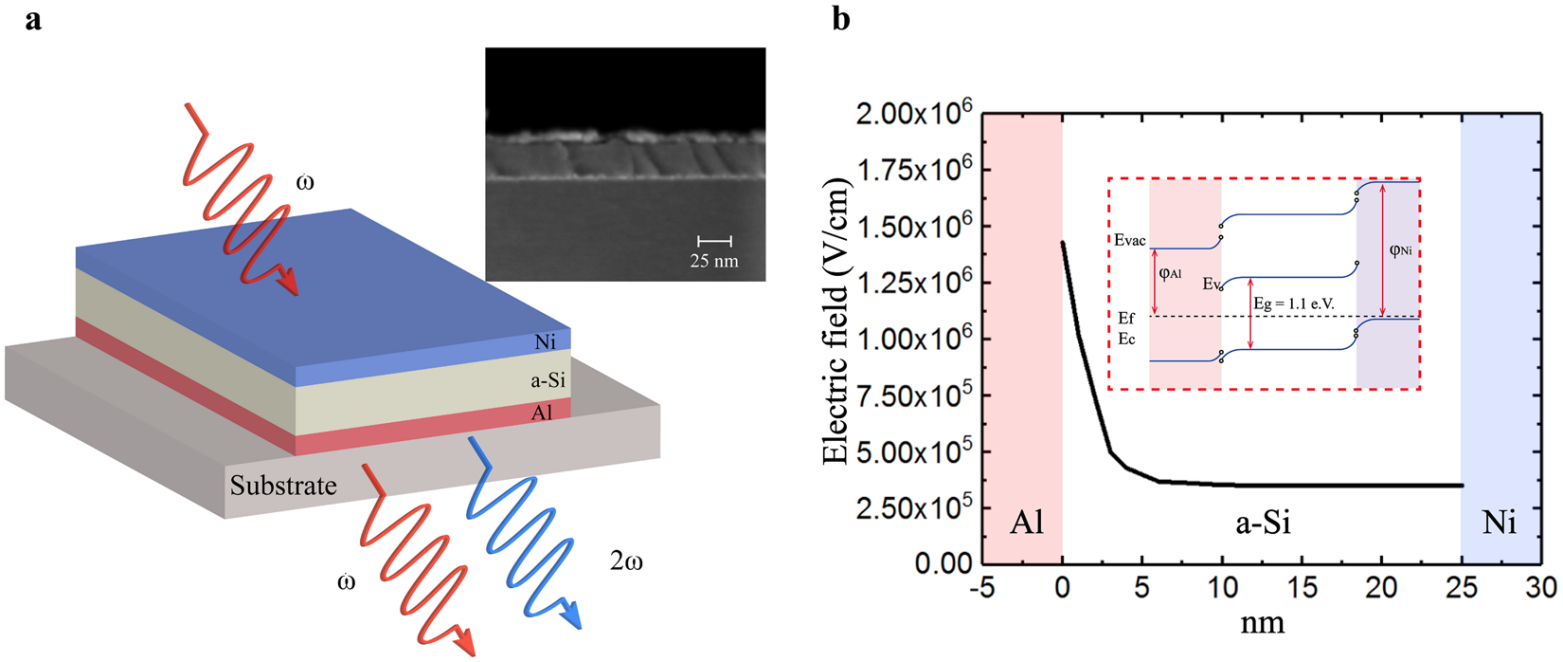
(**a**) Schematic and SEM image of a 5 nm Ni/25 nm a-Si/5 nm Al MSM metamaterial for second-harmonic generation process. (**b**) The reduced band diagram and the simulated distribution of built-in electric field for the Ni/a-Si/Al metamaterial.

The basic principle of the proposed MSM nonlinear optical metamaterial and the SEM image of the fabricated structure are shown in Fig. 1a, where a thin layer of a-Si is clad with Al and Ni which have respectively smaller and larger work function (i.e., Al with 4.08 eV and Ni with 5.01 eV)^26, 27^, compared to the work function of a-Si (i.e., 4.67 eV). Consequently, a static electric field is generated within the bulk of the a-Si semiconductor layer. The band diagram of the MSM structure is included as an insert in Fig. 1b showing a-Si’s energy band gap E_g_, Fermi level E_F_, valence and conduction bands energies E_V_ and E_C_ respectively. ϕ_Al_ and ϕ_Ni_, are the work function of Al and Ni respectively. As metals and a-Si are brought into contact, Al which has a lower work function causes diffusion of electrons into a-Si until thermal equilibrium is reached and the E_F_ of a-Si and Al align. The charge redistribution results in a built-in or diffusive potential, and creates a built-in static electric field close to the Al-a-Si interface, bending the bands of a-Si downwards. On the other side, once Ni with a larger work function is in contact with the a-Si layer, the heterostructure becomes asymmetric with the bands bent upwards close to the Ni-a-Si interface, resulting in an asymmetric built-in potential within the a-Si layer, and consequently a non-zero internal electric field, E_DC_. The difference in the metals’ work functions and the Fermi level of a-Si determines the depletion width and hence the magnitude and penetration depth of the induced electric field which can range from tens to hundreds of nanometers. With an appropriate choice of the two metals and a semiconductor layer thickness as narrow as the created depletion region, a high static electric field can be induced in the entire a-Si layer, leading to a high effective second-order nonlinear optical coefficient, χ^(2)^, via the EFISH effect.

We use TCAD Silvaco simulation tool^28^ to study the magnitudes and spatial distributions of the built-in electric fields inside the MSM metamaterial structures consisting of a 25 nm thick a-Si layer clad with 5 nm thick Al and Ni thin films (see Fig. 1b). As expected, the net electric field in the case of the MSM metamaterial is non-zero corresponding to an average DC electric field of 3.5 × 10^5^ V/cm. Since the a-Si layer exhibits a large third-order nonlinear susceptibility, χ^(3)^ (~2.3 × 10^−19^ to 9.2 × 10^−19^ m^2^/V^2^)^29–31^ the induced DC electric field is expected to result in a high effective χ^(2)^ = 3χ^(3)^E_DC_, ranging from 10 to 40 pm/V.

### Characterization of nonlinear coefficient in various MSM metamaterial compositions

For an experimental validation of our hypothesis, we designed and fabricated the MSM metamaterial on fused silica substrates using magnetron sputter deposition at room temperature (see Methods). We then experimentally estimate the induced χ^(2)^ coefficients in a-Si layer by measuring the generated SHG intensity and subsequently using the revised Maker fringes analysis described in details in the Supplementary materials^32, 33^.

**Figure 2:**
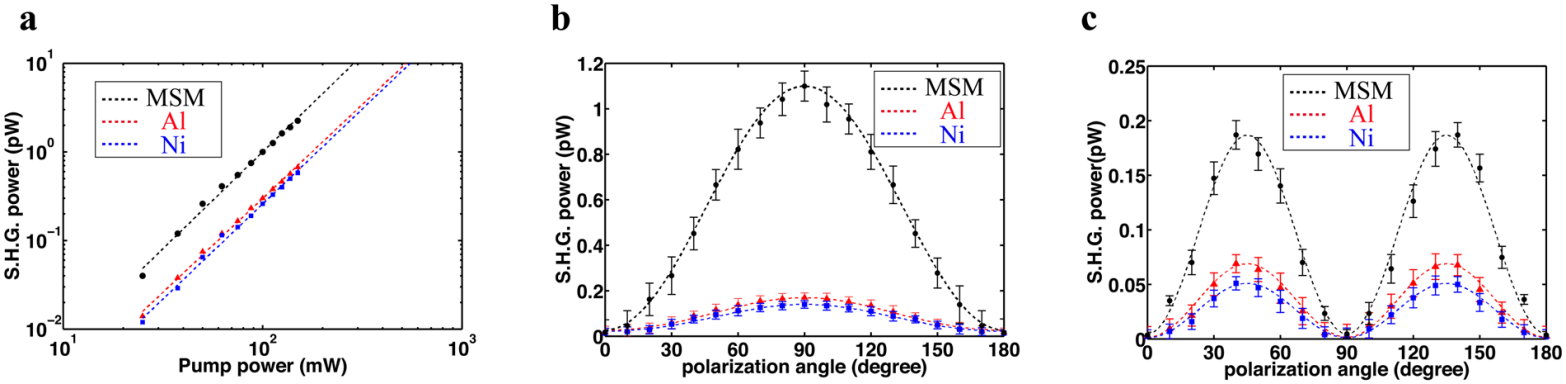
(**a**) The log-log plot of measured SHG signal versus pump power for a single layer of Al (red triangles), Ni (blue squares) film and MSM metamaterial (black circles) grown on silica substrates for a fixed pump-beam incident angle of 45°. (**b**) The generated p-polarized and (**c**) s-polarized SHG intensity versus polarization angle for a 100 mW pump.

First, to ensure that the detected signal is generated via the second-order nonlinear process from the bulk of deposited a-Si semiconductor, we analyze the measured signals as a function of the input pump power for both the MSM metamaterial as well as single layers of the cladding metals Al and Ni on fused silica substrates (see Fig. 2a). The measured intensities from all samples are confirmed, as expected, to be quadratically dependent on the pump power, illustrating the fact that the detected signals indeed originate from the second-order nonlinear responses. The a-Si film and the substrate are also confirmed to provide zero SHG signal as a result of their null χ^(2)^. We then apply Marker fringes method to characterize the polarization dependence of the SHG for extracting the χ^(2)^ tensor coefficients. The measured p- and s- polarized second-harmonic responses as a function of the polarization angle of the input pump are shown, for all the three cases, in Fig. 2b and c, respectively. For both polarizations, the SHG intensities from the MSM are clearly much larger than that generated from a combination of Al and Ni layers on fused silica substrates, implying that, as expected, the second-order nonlinear response is dominated by the a-Si layer due to the existence of the non-zero built-in electric field.

**Figure 3:**
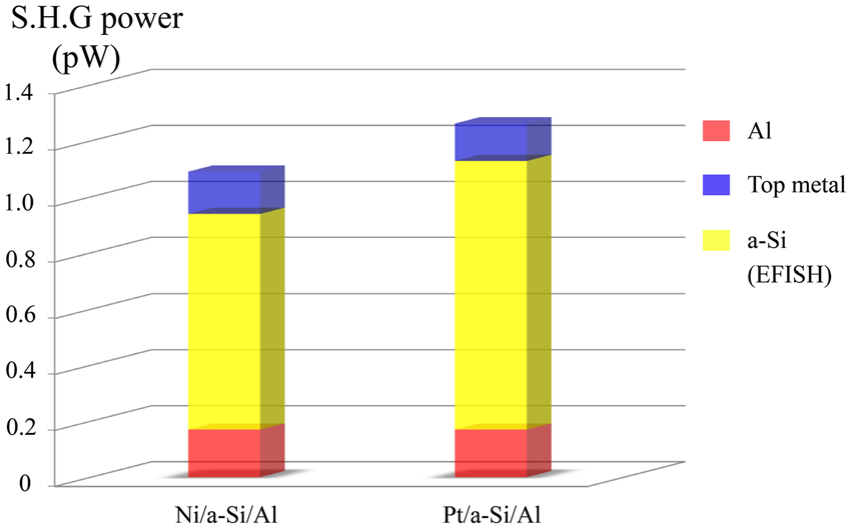
Measured SHG intensities from the Ni/a-Si/Al (left) and Pt/a-Si/Al (right) MSM metamaterials and their constituents. The red boxes show the SHG from a single layer of Al film, and the blue boxes show the SHG from top cladding metal films. The difference between the total measured SHG intensities from the MSM metamaterials and their components is then assumed to be from the bulk of the a-Si (yellow) layer via EFISH effect.

To further confirm our theory, we fabricated one more MSM metamaterial sample, replacing the top clad of Ni with platinum (Pt). Pt, just like Ni, has a larger work function (i.e., 5.65 e.V.)^26, 27^ than that of a-Si, and hence is expected to result in a non-zero built-in electric field in the a-Si layer in combination with the bottom clad of Al. The intensities of measured SHG from the Pt/a-Si/Al composition are measured and compared with the previous samples (i.e, Ni/a-Si/Al), as shown in Fig. 3, under the same experimental conditions. The SHG signal from the bottom Al cladding layer (red boxes) is fixed in all cases, and the signals from the top metal cladding films are measured individually and represented by blue boxes. The differences between the measured signals from the MSM metamaterials and their metallic constituents correspond to the contribution from a-Si layer (yellow boxes). It is apparent from Fig. 3 that the SHG signal in both MSM samples are significantly larger than the contributions from individual responses of their respective constituents. The generated second-harmonic field intensity can in fact be five times larger than the SHG signals from the respective constituents (e.g., individual Al, Ni and Pt metal films). Furthermore, and confirming our hypothesis, the contribution from the a-Si layer is found to be proportional to the difference of work functions between bottom and top metals, i.e., proportional to the built-in electric field. It can be envisioned that similar to the work demonstrated in this study, MSM metamaterials can, in the future, be custom-synthesized by choosing an appropriate combination of clad metals and semiconductor layer.

Additionally, the components of χ^(2)^ tensor in these MSM metamaterials are also estimated. We consider the thin MSM structures as a uniform isotropic effective medium^34^, and then calculate all the induced χ^(2)^ tensor components using the measured p- and s-polarized SHG signals for each of the MSM metamaterial samples (Supplementary Information). It should be noted that for more accurate theoretical estimate of the SHG via EFISH effect in our metamaterials one could consider optical field localization effects on each metal-semiconductor interface similar to these discussed in ref. 35. The results are summarized in the Table 1. Comparing the magnitude of all non-zero tensor components, it is apparent that the all-normal tensor component, *χ* ^*(2)*^_*zzz*_, which is parallel to the direction of the built-in electric field, is dominating, proving conclusively that the additional contribution to the second-order nonlinear response of an asymmetrical MSM metamaterial is truly a result of the engineered electronic band structure in the bulk of the a-Si layer.

**Table 1:** Calculated components of the effective χ^(2)^ tensors.

(pm/V)	*Ni/a-Si/Al*	*Pt/a-Si/Al*
*χ* ^*(2)*^_*zzz*_	7.2 ± 1.5	8.9 ± 1.6
*χ* ^*(2)*^_*xxz*_	1.9 ± 0.5	2.2 ± 0.8
*χ* ^*(2)*^_*zxx*_	2.3 ± 0.8	2.3 ± 0.7

Since our experiments were carried out using optical fields with photon energies larger than bandgap of a-Si, we need to consider the effect of generated free-carriers that may affect the magnitude of the induced effective χ^(2)^. The pump generated photocarriers could lower the Schottky barriers between the semiconductor and metals, and cause a reduction in the induced built-in electric field. To estimate the strength of this effect, we measured and analyzed the I-V behaviors of the MSM stack (see Supplementary material). These experiments show that the built-in barrier height within the a-Si layer, under illumination with an average power of 100 mW (i.e., the power used in our experiments on SHG), will be reduced by 15% compared to its value under no illumination. This reduction, resulting from the image-force effect as a consequence of the generated photocurrent, however results in a reduction of the built in electric field by no more than 10% for the average pump-powers employed in this study. Therefore, we conclude that the pump-generated photocarriers have a relatively minor effect on the measured effective χ^(2)^. It should be noted that the reported measured values of effective χ^(2)^ may increase by avoiding photocarrier generation using pump photon energies smaller than the bandgap of a-Si (e.g., 1550 nm range).

### Electrical control of MSM nonlinear response

**Figure 4:**
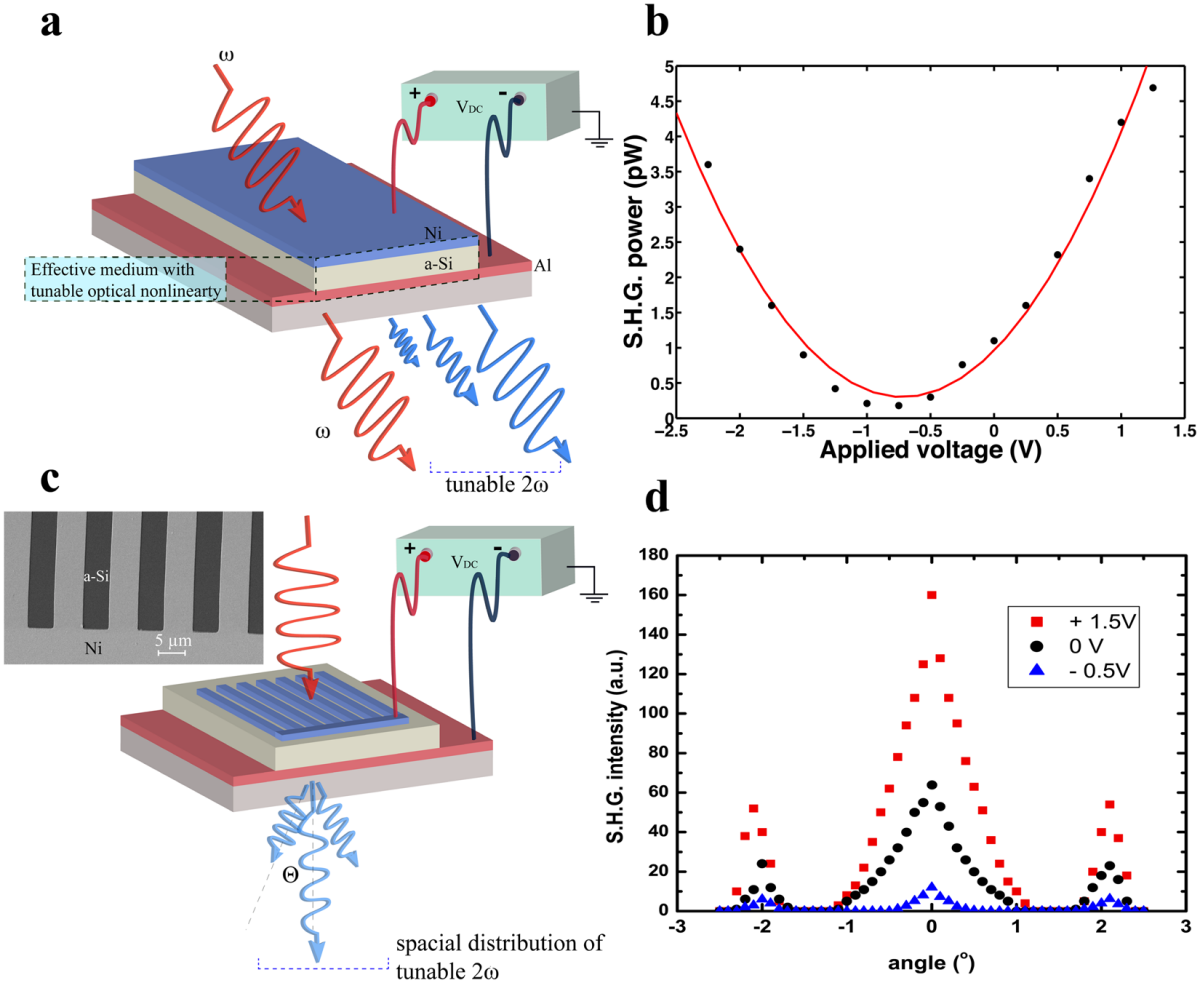
(**a**) Schematic of an active MSM metamaterial. (**b**) Measured SHG intensity under variant DC voltage (dots), and the red fitting curve showing the quadratic dependency. (**c**) Schematic representing the design for creating spatial nonlinear responses, and the top-view SEM image of the fabricated metal grating. (**d**) The SHG intensity from the patterned MSM structure detected under different angles and external electrical biases

In addition to their passive optical behavior, MSM metamaterials also enable the active control of their second-order nonlinear optical response by applying an external electric field using the constituent metals as electrodes. To illustrate this, experiments were carried out on a MSM metamaterial sample fabricated with a thicker a-Si layer of 50 nm, since the built-in electric field in the 25 nm thick a-Si layer is already close to the breakdown limit. The schematic of this active MSM structure is shown in Fig. 4a. An external DC voltage is applied across the a-Si layer, and the generated SHG signal is measured for bias voltages ranging from −2.5 V to +1.5 V. Figure 4b, shows the measured SHG intensity varying quadratically as a function of the applied field^16^, which is expected for a linear change in the second-order nonlinear susceptibility coefficient as a function of the applied bias. A positive voltage increases the generated SHG signal by inducing more asymmetry in band structure of the a-Si layer, until the built-in electric field reaches the breakdown limit, which in our case occurs at +1.5 V. For negative voltages, we observe a reduction in the SHG signal as a result of the reduction in the built-in electric field. At the flat-band voltage, which is negative in this case, the electric field inside the semiconductor is completely eliminated, removing any of its contribution to the observed second-harmonic response. It should be noted, that even at this voltage we observed a finite, non-zero, SHG signal, due to the two metal films. However, this is not the voltage corresponding to the absolute minimum observed second-harmonic signal. This, in fact, is achieved at a voltage of −0.75 V, where the generated second-harmonic field intensities from a-Si and the metals are out of phase and completely cancel each other. The measured intensity, however, still does not reach a zero value, which is attributed to the fact that the non-diagonal components of χ^(2)^ tensor still remain non-zero and result in a finite SHG signal. As the magnitude of the voltage applied is increased further, the SHG signal increases again until the breakdown field is reached, which occurs at −2.5 V. It is noteworthy that the induced effective dipoles in a MSM metamaterial under negative and positive fields should exhibit opposite directions even though the measured SHG signal shows the same magnitude. The minimum and maximum values of the measured SHG intensity are then used to determine that a wide tunable range of 2.8 to 15.6 pm/V is achievable for of the effective *χ* ^*(2)*^_*zzz*_ tensor in this MSM metamaterial.

In addition to these results, we also explore the possibility of spatially patterning one of the metal layers in our MSM structure, to electrically control the local spatial strength of the induced second-order nonlinearity. For simplicity, we pattern one of the electrodes into a periodic structure (i.e., grating), but more complex spatial structures with multiple control lines are indeed possible. A schematic diagram and the corresponding SEM image of the fabricated device are shown in Fig. 4c. The Ni film is patterned with a periodicity of 10 μm and a duty cycle of 0.5. We fabricate a total of 40 periods to ensure that the size of this pattern is larger than the spot size of our laser pump beam. Such a device exhibits alternating regions with different magnitudes of χ^(2)^. When a pump beam at frequency ω is incident on such a device, the generated second harmonic field will produce diffraction orders. Figure 4d shows the detected SHG signal exhibiting both 0^th^ and symmetrical ± 1^st^ diffraction orders. Furthermore, when an external voltage is applied across this patterned MSM structure, such that the applied electrical field is in phase (i.e., polarity) with the internal field, we obtain an enhanced diffraction efficiency (see Fig. 4d, red squares) whereas when the polarity is reversed, we obtain a corresponding reduction in the diffraction efficiency (see Fig. 4d, blue triangles) of the SHG signals. The tunability of the demonstrated spatially patterned second-harmonic response may enable novel optical signal processing applications with tailored space-invariant as well as space-variant nonlinear spatial impulse response.

## Conclusions

Asymmetrical metal-semiconductor-metal (MSM) electronic metamaterials exhibiting a prominent and tunable effective second-order nonlinear optical susceptibilities χ^(2)^ are introduced and experimentally validated. It is conclusively shown that a large induced χ^(2)^ originates from the interaction between the bulk third-order nonlinear optical susceptibility of the semiconductor layer, χ^(3)^ with the engineered non-zero net internal electric field created by the electron transport on its interfaces with metals possessing dissimilar work functions. The MSM structures, consisting of CMOS compatible a-Si layer clad with Al-Ni and Al-Pt, are shown to generate as much as five times larger second-harmonic field intensity compared to the contribution from its constituents. The nonlinear response of MSMs, proportional to the difference in the work functions of the cladding metals, is shown to be actively controlled by external electric field resulting in tunable effective *χ* ^*(2)*^_*zzz*_ tensor component ranging from 2.8 to 15.6 pm/V, making it suitable for on-chip optical switching and modulation. Further enhancement in the effective χ^(2)^ of the MSM metamaterials can be achieved by using a semiconductor possessing a larger χ^(3)^,with a larger breakdown voltage, and/or by improving the quality of a-Si fabrication process to increase its breakdown voltage. Moreover, by spatial patterning one of the metal layers on the interface with a-Si and varying the externally applied electric field, a tunable nonlinear diffraction effect is demonstrated, making the MSM metamaterial suitable for all-optical spatial signal processing with tailored space invariant as well as space variant nonlinear spatial impulse response.

## Methods

The metal/a-Si/metal metamaterials are grown at room temperature in an ultra-high vacuum environment by magnetron sputtering. Samples are deposited from Si-undoped (99.999%), Pt (99.95%), Ni (99.95%) and Al (99.95%) pure elemental targets onto fused silica substrates under an Ar pressure of 3 mTorr. Substrates undergo a thorough cleaning processes with Acetone, Isopropyl alcohol and deionized water before deposition, to prevent any inaccuracy in the optical characterization due to adhesion of particles on the surfaces. The thickness and refractive index of films are measured with the Rudolph Auto EL Ellipsometer. The deposition rate of each target and crystallization of films are characterized by means of small angle x-ray reflectivity (XRR) with a Bruker D8 Discover x-ray diffractometer. The XRR measurements, shown in the supplementary material of our previous work, confirm the fact that all sputtered films are amorphous^22^.

## Electronic supplementary material

Supplementary material
