# Supplementary material for: Electronic Metamaterials with Tunable Second-order Optical Nonlinearities
Source: Sci Rep. 2017 Aug 30;7:9983. doi: 10.1038/s41598-017-10304-2 (PMC5577114; doi:10.1038/s41598-017-10304-2)
Supplement: Supplementary file 1 — Supplementary material [file 41598_2017_10304_MOESM1_ESM.pdf]

Supplementary Information

Electronic Metamaterials with Tunable Second-order Optical Nonlinearities

Hung-Hsi Lin,[1†] Felipe Vallini,[2†] Mu-Han Yang,[2] Rajat Sharma,[2]
Matthew W. Puckett,[2] Sergio Montoya,[2,3] Christian D. Wurm,[2]
Eric E. Fullerton,[2,3] and Yeshaiahu Fainman[2*]

[1]*Materials Science and Engineering, University of California, San Diego, 9500 Gilman Drive, La Jolla, California 92093, USA*

[2]*Department of Electrical & Computer Engineering, University of California, San Diego, 9500 Gilman Drive, La Jolla, CA 92093, USA*

[3]*Center for Memory and Recording Research, University of California, San Diego, 9500 Gilman Drive, La Jolla, CA 92093-0401, USA*

[†]*These authors contributed equally to this work*

[*] *email: fainman@eng.ucsd.edu*

The contents of Supplementary Information include:

1. Optical characterization setup
2. Maker fringes analysis
3. Analysis of the effect of photocurrent on the built-in electric field within a-Si layer

Optical characterization setup

The optical characterization is carried out via the Maker fringe setup shown in Supplementary Fig.1. The pump beam is generated using a Ti:Sapphire laser emitting 150 fs pulses with a 80 MHz repetition rate at a center wavelength of 800 nm. The polarization state of the pump is defined by a half-wave plate and a long pass filter with cut-off wavelength at 780 nm is set to filter out any signals from other sources in the range of interest. The sample is tilted and fixed at an angle of 45 degrees normal to the incident beam, which is focused onto the sample surface using an 10x objective lens, resulting in a beam size with a radius of 20 μm. At the output, two short wavelength pass filters and one band pass filter with a total optical density of 12 are inserted to filter out the pump light at ω (i.e., 800nm wavelength), ensuring that all photons collected by the photomultiplier (PMT) are at 2ω (i.e., 400 nm wavelength) and consequently generated from the SHG process. The collimated SHG signal from the sample is separated into p-polarized and s-polarized by a polarizer for calculating the different components of the $\chi^{(2)}$ tensor. The detected signal in the PMT is then read with an oscilloscope. A commercial 500 μm thick X-cut quartz wafer, exhibiting a nonlinear coefficient $\chi^{(2)}_{xxx}$ of 0.64 ± 8% pm/V is used to calibrate the system,[1,2] and

the absolute values of $\chi^{(2)}$ tensor components from our samples are determined by comparing the generated SHG signals with those from the quartz sample under the same experimental conditions. Bare fused silica substrates are also characterized under the same conditions as samples with grown metamaterials to ensure that substrates do not contribute to any SHG signal. The second-harmonic response from a single layer of a-Si is found to be negligible compared to the large detected signals from the metal films and MSM metamaterials which is expected due to its amorphous nature. The measurement errors in our setup originate mainly from the fluctuation of laser power due to the varying humidity in the environment (±5%), background noises (±20%), and the non-uniformity in the thickness of the deposited thin films (±10%). In addition, the possibility of counting error (±10%) of photons in the PMT (Hamamatsu Inc., H11461-03) due to pulse-overlapping, as described in the handbook, is also taken into account. In order to minimize these errors, the generated SHG intensities from quartz, metal films and MSM metamaterials are determined by taking the average of those measured from five different spots on each sample. Following Herman's equation,[3] three tensor components, $\chi^{(2)}_{zzz}$, $\chi^{(2)}_{xxz}$ and $\chi^{(2)}_{zxx}$ can be extracted

by fitting the generated s- and p-polarized second-harmonic signals under various polarization angles of the incident pump beam.

Maker fringes analysis

Since a-Si is an amorphous material, we assume that its third-order susceptibility tensor $\chi^{(3)}$ components are same as those of an isotropic material with $C_{\infty,v}$ space symmetry and thus has 21 nonzero elements, of which only 3 are independent:

$$yyzz = zzyy = zzxx = xxzz = xxyy = yyxx \quad (1)$$

$$yzyz = zyzy = zxzx = xzxz = xyxy = yxyx \quad (2)$$

$$yzzy = zyyz = zxxz = xzzx = xyyx = yxxy \quad (3)$$

$$xxxx = yyyy = zzzz = zzxx + xzxz + xzzx \quad (4)$$

The existence of a static electric field in z direction is expected to introduce effective $\chi^{(2)}$ tensor components: $\chi^{(2)}_{xxz}$, $\chi^{(2)}_{xzx}$, and $\chi^{(2)}_{zxx}$ and $\chi^{(2)}_{zzz}$ through the EFISH effect. We assume that the MSM structures are isotropic in the transverse (i.e., in-plane) direction and that multiple reflections within the thin films can be neglected due to the thin nature of constituent films (i.e., 5 and 25 nm) compared to the wavelength of pump light (i.e., 800nm).[4] With the assistance of Maker fringes analysis,[3] these non-zero $\chi^{(2)}$ tensor components of the MSM metamaterial: $\chi^{(2)}_{xxz}$, $\chi^{(2)}_{zxx}$ and $\chi^{(2)}_{zzz}$ can be determined from fitting the generated s- and p- polarized second-harmonic signal intensities at frequency 2ω as a function of the polarization of the fundamental pump beam at frequency ω, measured at a fixed angle of incidence, θ. Also, since the

thicknesses of MSM metamaterials are much smaller than the wavelength of the pump beam, it is justified to use the effective medium theory for determining the three non-zero components of $\chi^{(2)}$ tensor[5,6] in all MSM metamaterial structures. By neglecting multiple reflections within each layer, the transmitted p- and s- polarized second- harmonic signals with energy of 2ω can be expressed as:[4]

$$P_{2\omega}^{s/p} = \frac{2\left((\sin(\phi))^2\left(t_{af}^{(p)}\right)^2 + (\cos(\phi))^2\left(t_{af}^{(s)}\right)^2\right)^2\left(T_{fs}^{(s/p)}\right)^2\left(T_{sa}^{(s/p)}\right)^2}{(n_2\cos(\theta_2))^2 cA\varepsilon_0} P_{1\omega}^2\left(\frac{2\pi}{\lambda}L\right)^2 d_{eff,s/p}^2$$
$$\times\, exp\left(-2(\delta_1+\delta_2)\right)\frac{(\sin\Psi)^2 + (\sinh X)^2}{\Psi^2 + X^2} \qquad (5)$$

Here, following Herman's notations, $P_{1\omega}$ and $P_{2\omega}$ denote the power at fundamental and second harmonic frequency, respectively, and the superscript s/p represents the state of polarization;[3] ϕ is the angle of incidence for fundamental frequency beam while ϕ at 0° and 90° represent s- and p- polarized respectively; *t* and *T* are Fresnel transmission coefficients for the fundamental field at frequency, ω and second harmonic field at frequency, 2ω, respectively; the subscripts *af*, *fs* and *sa* stand for interfaces between air (a), f (film) and s (substrate); c is the speed of light in vacuum; A is the area of the spot size of the fundamental beam focused on samples surfaces; ε_0 is the absolute permittivity of free space; L is the thickness of MSM structures; λ is the wavelength of incident fundamental laser beam, which is set at 800 nm in our

experiments. The incidence angle, θ_m inside MSM structure can be estimated using Snell's law $\sin\theta_m = \frac{1}{n_m}\sin\theta$ for the fundamental m=1 and second harmonic m=2 beams. Both a-Si and metals have non-zero absorption coefficients at the fundamental and second- harmonic fields. The complex index of refraction can be expressed as $\widetilde{n_m} = n_m(1 + i\kappa_m)$, where n_m denotes the real part of refractive index and κ_m represents the extinction coefficient of the materials at the frequency mω with m=1 and m=2 for the fundamental and second harmonic fields. Since the total thickness of MSM structures is small compared to incident wavelength, we use the effective medium approximation yielding the following expressions :[5,6]

$$n_{eff} = \sqrt{\frac{\sum_i^s n_i^2 h_i}{h}} \tag{6}$$

$$\kappa_{eff} = \sqrt{\frac{\sum_i^s \kappa_i^2 h_i}{h}} \tag{7}$$

where $i = 1,2,3, \ldots, s$, designate the composites in MSM structures, and h_i is the thickness of the i-th layer. The real and imaginary part of refractive index from different metals and a-Si are measured with Rudolph Auto EL Ellipsometer, yielding values in Supplementary Table 1.

Following quantities shown in Supplementary equation (5) represents the influences of imaginary part of refractive index on generated SHG intensity for absorbing nonlinear materials. The physical meaning can be found in details from Herman's works:[4]

$$\delta_m = \frac{2\pi L}{\lambda}\frac{n_m \kappa_m}{\cos\theta_m} \tag{8}$$

$$X = \delta_1 + \delta_2 \tag{9}$$

$$\Psi = \frac{2\pi L}{\lambda}(n_1\cos\theta_1 - n_2\cos\theta_2) \tag{10}$$

The relation between effective susceptibility $d_{eff,s/p}$ and three $\chi^{(2)}$ tensors is expressed as:

$$d_{eff}^{p} = -\frac{1}{2}(\chi_{xzx}\cos\theta_2\sin 2\theta_1(\sin\phi')^2 + \chi_{zxx}\sin\theta_2\,((\cos\theta_1\sin\phi')^2 + (\cos\phi')^2) + \chi_{zzz}\sin\theta_2\,(\sin\theta_1\sin\phi')^2) \tag{11}$$

$$d_{eff}^{s} = -\frac{1}{2}\chi_{xzx}\sin\theta_1\sin 2\phi' \tag{12}$$

The polarization angle in the thin film stacks ϕ' can be expressed by:

$$\tan\phi' = \tan\phi \times \frac{t_{af}^{p}}{t_{af}^{s}} \tag{13}$$

The detected s- and p-polarized SHG intensities are used to determine the effective susceptibility $d_{eff,s/p}$ by fitting measured signals under variant polarization states of incident fundamental beam. Based on Supplementary equation (11)-(13), absolute value of three components of $\chi^{(2)}$ tensor can be extracted in the bulk of all MSM metamaterials.

Analysis of the effect of photocurrent on the built-in electric field within a-Si layer

The induced effective $\chi^{(2)}$ in our MSM structures comes from the engineered non-zero static electric field within the semiconductor (i.e., a-Si) layer. Since the SHG measurements were carried out using optical fields with photon energies (i.e., 1.55 e.V.) larger than bandgap of a-Si (i.e., 1.1 e.V.), we need to consider the effect of light induced free carrier generation, which may affect the magnitude of the built-in electric field in a-Si, and, consequently, the induced effective $\chi^{(2)}$. In order to quantify the influence of the generated photocurrent on the built-in electric field, we performed I-V measurements (see Supplementary Fig. 2(a)) with and without illumination at the pump wavelength on the sample with the MSM structure consisting of Ni/a-Si/Al.

An external voltage V is applied across patterned 1mm x 1mm Ni metal squares and bottom Al metal film. The pump beam is focused on the patterns with a 10x objective lens resulting in a spot size with radius of 20 μm. The I-V characteristics are then studied under variant illumination conditions with a Keithley model 2400. Pump light with average power ranging from 12.5 to 200 mW is illuminated on the MSM structure and the I-V characteristics are shown in Supplementary Fig. 2 (b). By

analyzing the I-V curves, electrical properties such as the serial resistance of the MSM structure, dark current, photocurrent and the Schottky barrier height between metals and a-Si can be calculated.[7,8] These results are summarized in Supplementary Table 2.

The serial resistance of the MSM stack is barely affected by the laser beam in our experiments. The measured dark current in the MSM structure is determined to be 43.8 nA, whereas the photocurrent can be an order of magnitude larger than the dark current. The measured photocurrent is 401 nA when illuminated by a pump beam with an average power of 100 mW, identical to the condition of our experiments. This large induced photocurrent could lower the Schottky barrier height through image force effect,[9] in other words, lower the built-in electric field within a-Si, hence affecting the induced optical nonlinearity and thereby lead to an inaccuracy in the estimated values of $\chi^{(2)}$ tensors. Comparing the Schottky barrier height under dark to that under illumination with a power of 100 mW, we observed a reduction by 15%, confirming the fact that the image force effect does indeed lower the barrier in our case. We then simulate the influence of this effect on the built-in electric field using Silvaco tool, and determine that the decrease in the value of the induced field due to

the generated photocurrent is about 10%. In summary, we conclude that the pump-generated photocarriers have a minor effect on the measured effective $\chi^{(2)}$ in our MSM structure.

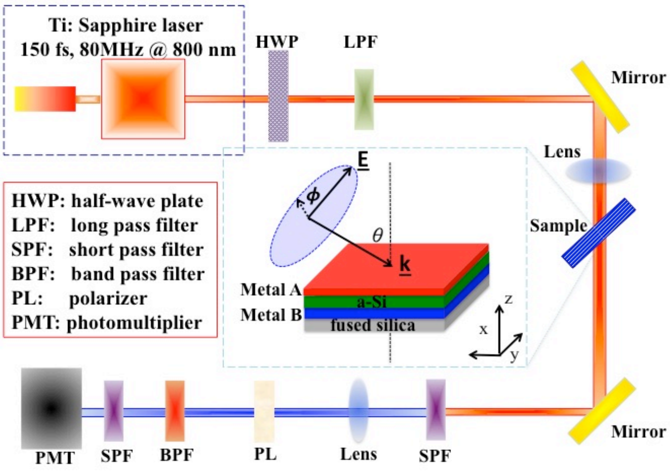

Supplementary Fig.1 Schematic of Maker fringes setup for optical characterization.

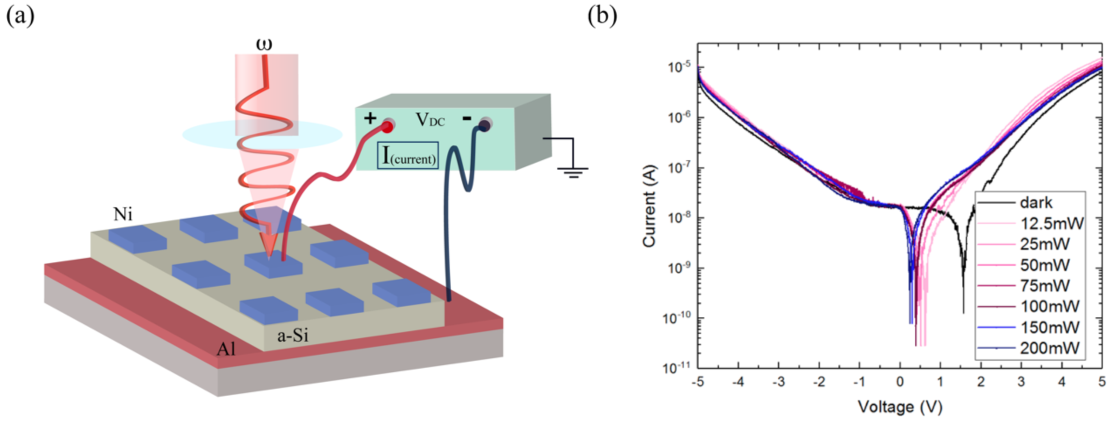

Supplementary Fig. 2(a) Schematic of the patterned MSM stack for measurement of I-V behaviors under different illumination conditions. (b) I-V behaviors without (black) and with illumination under variant of average power.

Supplementary Table 1. Real and imaginary part of refractive index for metal and a-Si thin films measured using elliposometer at 400 nm and 800 nm.

	Refractive index at 800nm	Refractive index at 400nm		Refractive index at 800nm	Refractive index at 40nm
Silica	n=1.46 κ=0	n=1.44 κ=0	Pt	n=2.61 κ=5.2	n=1.83 κ=2.1
a-Si	n=3.93 κ=0.09	n=4.3 κ=1.87	Ni	n=1.35 κ=2.8	n=2.42 κ=4.41
Al	n=2.68 κ=7.81	n=0.43 κ=4.42			

Supplementary Table 2. Electrical properties of a-Si layer under variant illumination conditions.

	Serial resistance (Ohm)	Dark current (nA)	Photocurrent (nA)	Schottky barrier height (V)
Dark	4.629 k	43.8	-	0.69
50mW	4.612 k	-	193	0.63
100mW	4.609 k	-	401	0.61
150mW	4.560 k	-	595	0.6
200mW	4.520 k	-	812	0.59

Supplementary References:

1. Sanford, N. A. et al. Measurement of second order nonlinear susceptibility of GaN and AlGaN. *J. Appl. Phys.*, **97,** 053512 (2005).
2. Shoji, I., Kondo, T., Kitamoto, A., Shirane, M., & Ito, R. Absolute scale of second-order nonlinear-optical coefficients. *J. Opt. Soc. Am. B* **14,** 2268-2294 (1997).
3. Herman, W. N. & Hayden, L. M. Maker fringes revisited: second-harmonic generation from birefringent or absorbing materials. *J. Opt. Soc. Am. B* **12,** 416-427 (1995).
4. Hermans, A. et al. On the determination of $\chi^{(2)}$ in thin films: a comparison of one-beam second-harmonic generation measurements methodologies. *Sci. Rep.* **7,** 44581 (2017).
5. Smalley, J., Vallini, F., Shahin, S., Kanté, B., & Fainman, Y. Gain-enhanced high-k transmission through metal-semiconductor hyperbolic metamaterials. *Opt. Mater. Express* **5,** 2300 (2015).
6. Haija, A., Freeman, W. & Roarty, T. Effective characteristic matrix of ultrathin multilayer structures. *Optica Applicata* **36,** 39-50 (2006).
7. Schmitsdorf, R. F., Kampen, T. U. & Mönch, W. Explanation of the linear correlation between barrier heights and ideality factors of real metal-semiconductor contacts by laterally nonuniform Schottky barriers. *Journal of Vacuum Science & Technology B: Microelectronics and Nanometer Structures Processing, Measurement, and Phenomena* **15,** 1221-1226 (1997).

8. Wagner, L. F., Young, R. W. & Sugerman, A. A note on the correlation between the Schottky-diode barrier height and the ideality factor as determined from IV measurements. *IEEE Electron Device Letters* **4,** 320-322 (1983).

9. Andrews, J. M. & Lepselter, M. P. Reverse current-voltage characteristics of metal-silicide Schottky diodes. *Solid-State Electronics* **13,** 1011-1023 (1970).
